# Alpha Reactivity Dynamics Reveal Multi-System Variability Without Global Alpha Slowing in Fragile X Syndrome

**DOI:** 10.21203/rs.3.rs-10362875/v1

**Published:** 2026-07-29

**Authors:** Shelby Dauterman, Lisa A. De Stefano, Elizabeth Smith, Eva McCord, Sebastian Piloto, Grace Westerkamp, Christina Gross, Ernest V. Pedapati, Makoto Miyakoshi, Craig A. Erickson

**Affiliations:** 1. Cincinnati Children’s Hospital Medical Center, Cincinnati, OH, USA; 2. University of Cincinnati, Cincinnati, OH, USA

**Keywords:** Fragile X Syndrome, Alpha Reactivity, Cortical Inhibition, Network Dysfunction, Sex Differences, EEG Biomarkers

## Abstract

Fragile X syndrome (FXS) is associated with alterations in alpha-band activity recorded using electroencephalography (EEG), including reports of alpha slowing, that may reflect impaired inhibitory regulation and network-level dysfunction. However, it remains unclear whether these abnormalities represent a global shift in oscillatory frequency or dissociable disruptions across distinct systems. In the present study, we leveraged alpha reactivity dynamics, defined as eyes-open to eyes-closed modulation, to probe alpha function in 23 individuals with FXS and 23 typically developing controls using both absolute and relative spectral measures. In FXS, absolute alpha reactivity showed a robust, region-specific reduction localized to occipital cortex, consistent with impaired sensory-inhibitory modulation. While relative alpha reactivity revealed more widespread attenuation across regions, indicating broader network alterations. Peak reactivity frequency showed no evidence of global slowing but was consistent with localized shifts toward lower frequencies. These effects were region- and sex-specific, with females showing reduced frontal relative peak frequency and males showing associations between lower relative peak frequency in non-occipital regions and higher IQ. These findings support a multi-system model of alpha dysfunction in FXS, characterized by posterior inhibitory deficits and distributed network alterations, and highlight alpha reactivity dynamics as a promising translational biomarker.

## Introduction

Fragile X syndrome (FXS), the most common inherited monogenic cause of intellectual disability, results from transcriptional silencing of the Fragile X Messenger Ribonucleoprotein 1 (FMR1) gene and consequent loss of its protein product, FMRP [1,2]. Because FMRP serves as a key translational regulator, its absence leads to excessive and dysregulated protein synthesis, particularly within the developing brain. This disruption interferes with critical neurodevelopmental processes, including neurogenesis, synaptic maturation, and activity-dependent pruning of neural circuits, ultimately producing widespread network-level abnormalities[2,3]. Clinically, these alterations manifest as delayed cognitive development, reduced intellectual functioning, and behavioral features such as anxiety, attention deficits, social avoidance, and sensory hypersensitivity[4,5]. FXS affects approximately 1 in 4000 males and 1 in 6-8000 females[6], yet no FDA-approved pharmacological treatments currently exist. The severity of FXS features and the persistent lack of effective therapies underscore the need for reliable translational measures and a deeper mechanistic understanding of FXS, particularly through characterization of network-level alterations that disrupt large-scale cortical signaling.

To address this translational gap, our lab has established resting-state electroencephalography (EEG) biomarkers. This approach has allowed us to characterize fundamental neurophysiological abnormalities in FXS, such as increased high-frequency gamma power[7,8], reduced inter-trial phase coherence to auditory stimuli[9,10], and a reduction in the brain’s peak frequency from the alpha range to the theta range [7,11–13]. Given the strong link between alpha oscillations and overall brain function, including their role in cortical inhibition, cognitive regulation, network communication, and the maintenance of stable brain states[14–17], characterizing how alpha activity is altered in FXS is essential for refining biomarker specificity.

Prior EEG studies in FXS found increased relative and absolute theta power, reduced relative alpha power, and indications that peak alpha frequency may shift toward slower frequencies or reflect increased theta-range influence (“alpha slowing”) [7,11,13]. This pattern has often been attributed to altered thalamocortical contributions that may disrupt the normal pacing of alpha activity, thereby elevating low-frequency activity and impairing regulation of cortical excitability[7,8,18]. Although it could not be determined whether this effect was causal or compensatory, this observation led to the proposal of thalamocortical dysrhythmia (TCD) as a unifying phenomenon that may account for certain EEG features observed in FXS [7,11]. However, this framing risks implying that alpha activity constitutes a single unitary phenomenon in FXS, whereas it is more accurately understood as comprising multiple functionally distinct components. Importantly, scalp-recorded alpha activity reflects contributions from two partially distinct generators that directly map onto the functional and mechanistic distinctions relevant to FXS. The classical posterior “inhibitory” alpha rhythm, which is maximal over occipital electrodes, is believed to arise primarily from cortico-cortical networks that regulate sensory-inhibitory control[15,17,19]. In contrast, alpha activity expressed over frontal, central, and temporal regions is thought to reflect thalamo-cortical propagation involved in cognition, arousal regulation, executive processing, and the timing of large-scale cortical operations[15,20,21]. This distinction is translationally meaningful in FXS, as different networks reflect different molecular alterations in FXS. For example, posterior cortico-cortical circuits are especially vulnerable to synaptic maturation and pruning deficits caused by FMRP loss[3,22–24], while thalamo-cortical pathways more directly reflect altered excitability and rhythmic pacing consistent with TCD[7,8,18,25].

A defining feature of alpha is its strong, reliable modulation during transitions between eyes-open (EO) and eyes-closed (EC) states[26,27]. This change in alpha power between conditions, termed “alpha reactivity,” provides a physiologically grounded index of how effectively the brain switches between externally oriented (exteroceptive) and internally oriented (interoceptive) modes of processing via inhibitory control[28]. For example, an increase in posterior alpha activity during EC reflects active inhibition of visual cortex, downweighing sensory inflow and promoting internally guided processing; conversely, alpha suppression during EO indicates a release of inhibition in sensory regions, facilitating perceptual engagement and exteroceptive processing. Thus, the magnitude of alpha reactivity captures the efficiency with which inhibitory gating mechanisms coordinate state-dependent transitions[29–31].

Within this framework, alpha reactivity offers a controlled approach to probing whether alpha band abnormalities in FXS reflect a unitary disruption or arise from multiple, dissociable mechanisms. Specifically, it allows differentiation between reduced inhibitory modulation; altered oscillator timing, as in the TCD framework; or their combined contribution. Examining a broader frequency range provides an assumption-free method for characterizing EO/EC alpha band dynamics in FXS, avoiding a priori constraints that maximal modulation must occur within the canonical alpha band (8–13 Hz). This approach allowed us to consider: (1) is alpha reactivity reduced in FXS relative to TDC, consistent with a global weakening of alpha modulation, or does it reflect selective disruption of specific alpha-regulatory mechanisms? (2) do distinct patterns of reactivity across cortical regions demonstrate that alpha dysfunction in FXS reflects disruption of multiple alpha generators and functions rather than a single, unitary disturbance? (3) does alpha reactivity slow globally in FXS, or diverge across functionally distinct alpha systems with different roles? (4) do these effects differ by sex, given known sex-linked variability in FMRP expression and behavioral phenotypes?

## Methods

This study was approved by the Cincinnati Children’s Hospital Medical Center Institutional Review Board, and all participants or their legal guardians provided assent and written informed consent in accordance with the Declaration of Helsinki.

### Participants.

Twenty-seven individuals with FXS (8 female; mean age = 29.82 years, SD = 7.72, range = 17–44) and 23 typically developing control participants (TDC; 9 female; mean age = 27 years, SD = 7.43, range = 15–43) were recruited through the Cincinnati Children’s Hospital Medical Center Fragile X Research and Treatment Center. Participants were required to be between the ages 15-45 years old. Participants with FXS had confirmed diagnoses via Southern Blot and PCR and were excluded if they had a seizure within the last 6 months or were taking benzodiazepines. TDC were excluded if they had a history of, or a first-degree relative with, any neurodevelopmental disorder, or if they scored ≥ 8 on the Social Communication Questionnaire (SCQ). At the time of participation, individuals with FXS were taking a range of psychotropic medications commonly prescribed in this population, including selective serotonin reuptake inhibitors (SSRIs), antipsychotic medications, stimulant and non-stimulant medications for attention-deficit/hyperactivity disorder (ADHD), and other medications. Medication was permitted with the exception of benzodiazepines.

After reviewing video collected during the task, four individuals with FXS were excluded due to noncompliance with task instructions or failure to obtain at least 30 seconds of clean data as noted by study staff. This resulted in a final sample of 23 participants with FXS (8 female; mean age = 30.18 years, SD = 7.99, range = 17–44) (Table 1).

### Clinical Measures.

To quantify overall cognitive and intellectual functioning, participants completed the abbreviated Stanford–Binet, 5th Edition (SB5; [32]). Standard scores were converted to deviation IQ, which provides a standardized estimate of global intellectual ability while reducing floor effects commonly observed in individuals with neurodevelopmental disabilities[33]. IQ served as the primary behavioral correlate with EEG features.

### FMRP Quantification.

Whole blood samples were spotted onto QIAcard Bloodstain cards in 50 uL spots, which were then processed through a highly sensitive and reproducible Luminex-based immunoassay developed by previous members of our lab[34]. FMRP was then quantified using a 12-point standard curve of human recombinant FMRP (GST-SR7 fusion protein, Institute for Basic Research in Developmental Disabilities), with a detection limit ranging from 0.03 to 70 picomolar (pM) units. To reduce the influence of outlying technical replicates, the FMRP value reported is a 20%-trimmed mean of protein concentration in pM.

### EEG Recording.

Continuous resting-state EEG was collected using a Phillips/EGI Net Amps 400 system (Eugene, Oregon, USA) with a 128-channel HydroCel saline-based electrode net, sampled at 1000 Hz. As previously published, EO EEG was recorded for 5–10 minutes while participants passively watched a silent video to improve compliance[7,11,13,35]. Impedances were kept below 50 kΩ, with Cz used as the reference electrode. Immediately after EO acquisition, participants were instructed to close their eyes, and a 2-minute EC resting-state EEG was obtained without adjusting impedances. Behavioral support, including video monitoring and staff guidance, was provided to encourage eye closure and minimize movement. EO contamination within the EC period was quantified using synchronized video and staff notes; datasets were excluded if EO activity comprised ≥ 20% of the EC recording, contributing to the exclusion of the four males with FXS mentioned above.

### EEG Preprocessing.

EEG data were blinded to participant diagnosis and sex prior to preprocessing in EEGLAB[36]. EO and EC recordings were concatenated, resampled to 250 Hz, high-pass filtered at 1.5 Hz, and notch filtered between 57–63 Hz. Artifact correction was performed using artifact subspace reconstruction (ASR)[37–39], which applies a sliding-window principal component analysis to detect and attenuate transient, high-variance artifact activity using thresholds derived from clean reference data. Channels excluded during reference data estimation, along with Cz, were subsequently interpolated, and the data were re-referenced to the average of all electrodes.

### Spectral Feature Extraction.

**Figure 1.**
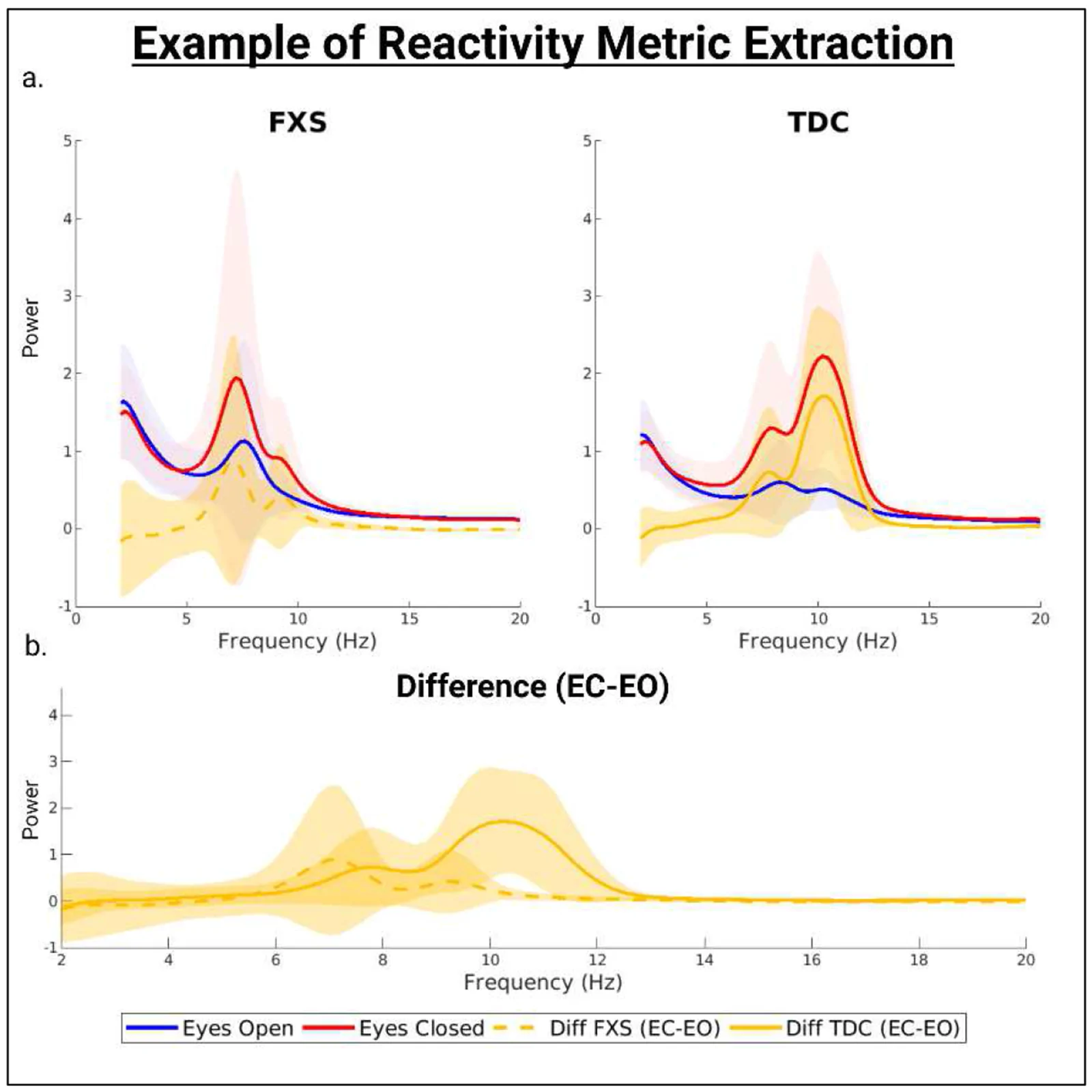
Schematic illustration of alpha reactivity metric extraction from EEG power spectra. (a) Example power spectral density (PSD) plotted for eyes-open (EO) and eyes-closed (EC) conditions. (b) Reactivity spectrum calculated as the EC–EO difference in PSD across frequencies. Peak reactivity was defined as the maximum positive value within the reactivity spectrum, and peak reactivity frequency was defined as the frequency at which this maximum occurred within the 4–13 Hz range. Shaded regions represent 95% confidence intervals around the mean PSD.

Power spectral density (PSD) estimates were generated for each participant, condition, and channel using a short-time Fourier transform implemented via MATLAB’s spectrogram function. Signals were analyzed using a sliding window equal to half the sampling rate with 50% overlap, and PSD was computed across frequencies from 2 to 100 Hz in 0.2 Hz increments. Median power across time was calculated at each frequency for a more robust approach against outlier contamination, and PSD values were retained in linear units (μV^2^/Hz). Theta (4–7.8 Hz) and alpha (8–13 Hz) bands were defined a priori using fixed frequency boundaries. The upper theta limit was extended to 7.8 Hz to ensure continuous spectral coverage across the conventional theta–alpha transition. Frequency resolution in the analysis was 0.2 Hz, which determined the exact placement of the upper band boundary. The resulting spectra reflect the absolute power at each frequency in each condition (EO and EC). To generate estimates of relative power, the absolute power at each frequency was normalized by the total power separately for each condition (Figure 1a).

For both absolute and relative power, we first computed the difference spectrum by subtracting the EO PSD from the EC PSD at each frequency, yielding the effect of eye closure across the spectrum (Figure 1b), consistent with prior EO/EC work in neurodevelopmental disorders, including autism[40]. Alpha reactivity was calculated as the mean EC–EO difference within the alpha band (8–13 Hz) and calculated separately for absolute (“absolute alpha reactivity”) and relative (“relative alpha reactivity”) power (Figure 1b), allowing condition-dependent changes to be evaluated in both raw and normalized forms. To identify the frequency of maximal reactivity without imposing assumptions about its expected locus, the EC–EO difference spectrum was evaluated over 4-13 Hz (Figure 1b), and the largest positive peak was extracted using MATLAB’s findpeaks() function. This procedure yielded the frequency that was most affected by eye closure (“relative peak frequency,” “absolute peak frequency”) as well as the magnitude of the reactivity (“relative peak reactivity,” “absolute peak reactivity”). This data-driven approach allowed the dominant reactivity frequency to emerge empirically for each participant rather than restricting analyses to the alpha band.

### Regions of Interest.

Regions of interest (ROIs) were defined using established HydroCel labels. Occipital electrodes included E70, E71, E75, E76, and E83; temporal electrodes included E41, E45, E46, E47, E52, E92, E98, E102, E103, and E108; frontal electrodes included E3, E11, E19, E23, E24, E28, E117, and E124; and central electrodes included E13, E36, E37, E55, E87, E104, and E112. ROI selection followed conventions from prior work in FXS for all regions except the posterior scalp, where earlier studies reported a broader posterior region rather than a strictly occipital subset[41]. To focus more directly on visual cortex–proximal sites, we adopted a more focal occipital ROI (as listed above) in place of the posterior composite used previously.

### Statistical Analyses.

All EEG metrics were exported to R (version 4.5.2; CITE) for statistical modeling. Linear mixed-effects models (lme4, version 2.0-1)[42] were fit with Group (FXS vs. TDC), Sex (male vs. female), and ROI (frontal, central, temporal, occipital) as fixed effects and participant as a random intercept. All post-hoc contrasts were conducted using *emmeans* (version 2.0.3)[43] package with Tukey correction for multiple comparisons. Figures were created utilizing ggplot2 (version 4.0.3)[44] and Biorender[45].

Exploratory clinical correlations were conducted to examine associations between clinical measures and study variables. Normality of continuous variables was assessed using the Shapiro–Wilk test. For pairs of variables that were approximately normally distributed, Pearson’s product–moment correlation coefficient (r) was used. When one or both variables deviated from normality, Spearman’s rank-order correlation coefficient (ρ) was applied. Given the small pilot sample size and the hypothesis-generating, exploratory nature of this study, correlation analyses were not corrected for multiple comparisons. Correlations between EEG variables and FMRP were examined in both the full sample with available FMRP data (n=19, 8 female) and separately in a subset of individuals with detectable FMRP expression (n=15, 8 female, > 0.03, our assay’s detection limit).

## Results

### Absolute Power

#### Absolute Alpha Reactivity.

**Figure 2.**
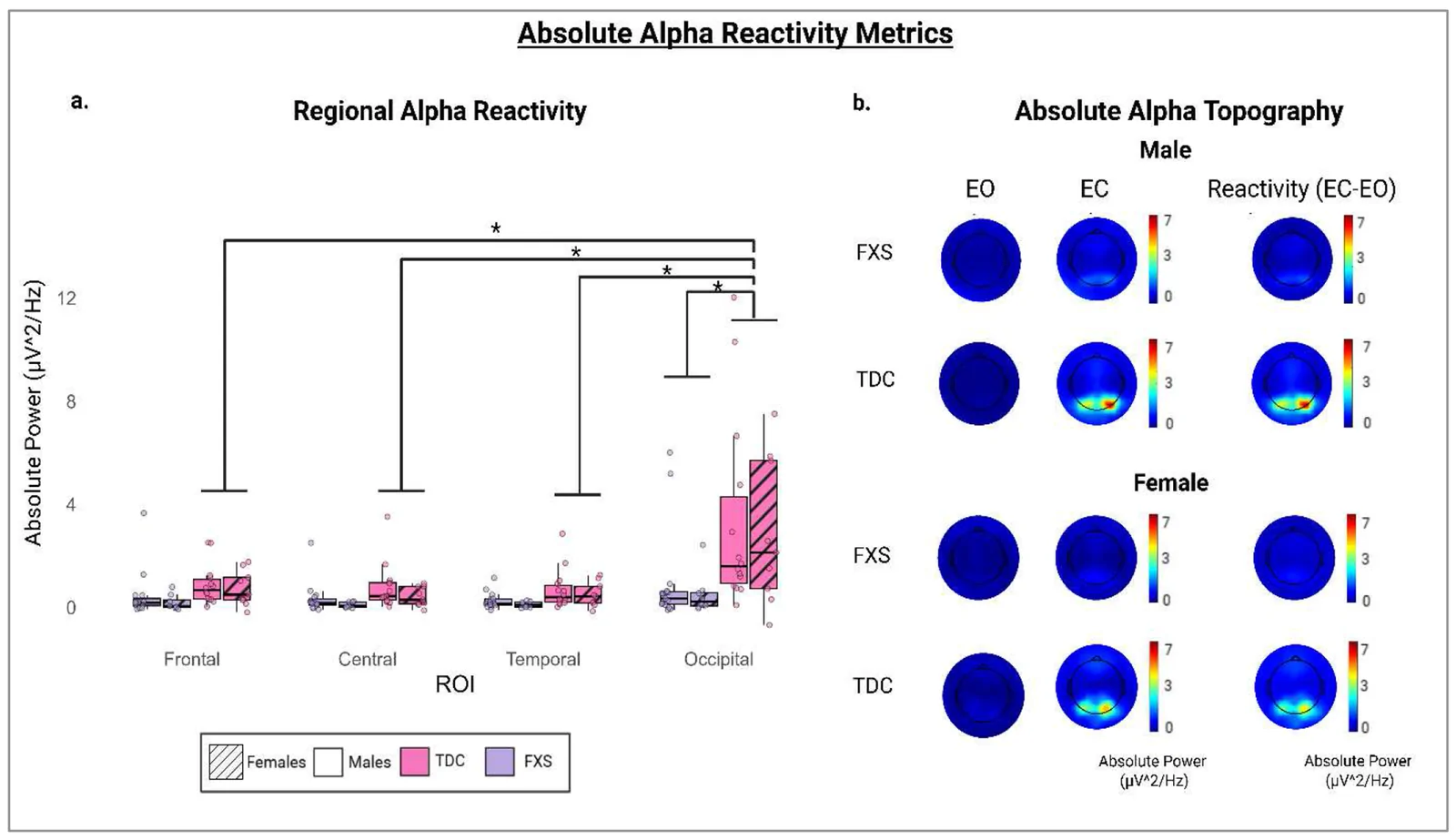
Absolute alpha reactivity (8–13 Hz; EC–EO) displayed as (a) a bar graph, shown separately for each region and group, (b) topographical plots showing the distribution of absolute alpha reactivity more granularly across the scalp. Boxplots display the median, interquartile range (IQR), with whiskers extending to 1.5× IQR; individual data points are overlaid. In both plots, TDC exhibit a pronounced posterior maximum, while FXS shows reduced occipital reactivity and a flattened topographical profile. A significant Group × ROI interaction reflects a selective reduction in occipital reactivity in FXS. *p < 0.05.

Analysis of absolute power-derived alpha reactivity revealed significant main effects of group (*F*(1, 42) = 7.16, *p* = 0.01) and ROI (*F*(3, 126) = 19.29, *p* < 0.001) as well as a significant Group × ROI interaction, *F*(3, 126) = 7.92, *p* < 0.001). In contrast, sex did not contribute meaningfully to the model. Together, these findings demonstrate that absolute alpha reactivity was reduced in some regions in a group dependent manner, with no detectable sex differences (Figure 2a).

Within the FXS group, no pairwise differences between ROIs reached significance (all |*t*| ≤ 1.71, all *p* ≥ 0.32), suggesting a relatively flat or undifferentiated topography. Thus, absolute alpha reactivity in FXS did not show reliable distinctions across frontal, central, temporal, or occipital sites (Figure 2b). In contrast, the TDC group displayed a clearly structured topographical pattern. Absolute alpha reactivity was significantly greater in the occipital region than frontal (*t* = −6.85, *p* < .0001), central (*t* = −7.46, *p* < .0001), and temporal regions (*t* = −7.50, *p* < .0001), whereas non-occipital regions did not differ significantly from each other (all *p* ≥ 0.92) (Figure 2a). This pattern reflects a well-defined posterior peak in absolute alpha reactivity in TDC that was not present in FXS**.**

Between-group testing conducted at each ROI further revealed spatial differences manifested across diagnostic groups. Group differences were absent at frontal, central, and temporal regions (all |*t*| ≤ 1.08, all *p* ≥ 0.28), indicating comparable levels of absolute alpha reactivity between groups for these regions. In contrast, a robust group effect was observed in the occipital region, where FXS showed significantly lower reactivity than TDC (*t* = −5. 22, *p* < 0.0001) (Figure 2a).

#### Absolute Peak Frequency.

**Figure 3.**
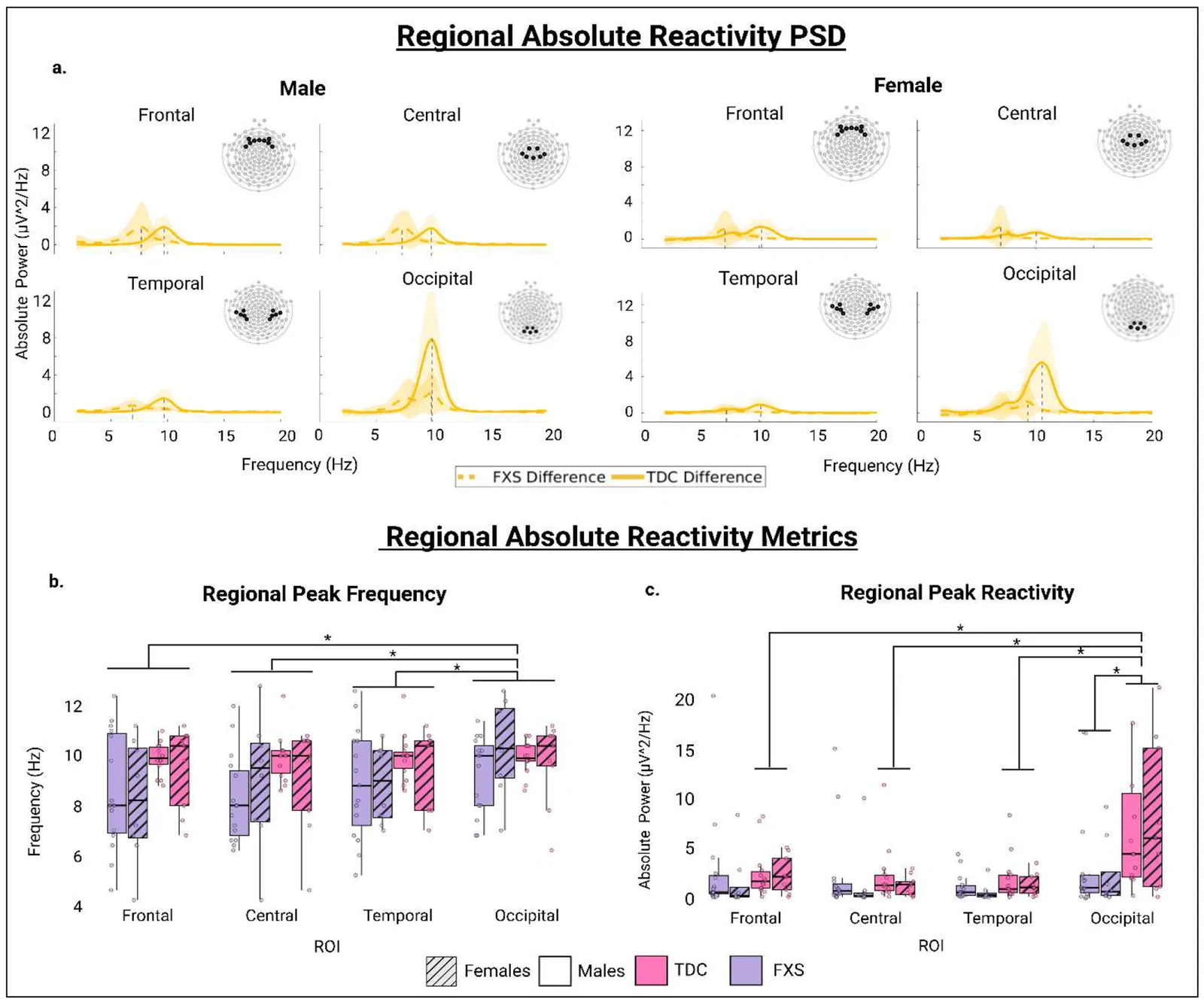
Absolute reactivity metrics. Representative PSD plots from cleaned EEG data (a). Absolute peak reactivity magnitude and peak reactivity frequency (identified within 4–13 Hz) across regions in FXS and TDC (b, c). Boxplots show the median, IQR, and 1.5× IQR whiskers with individual data points overlaid. Peak reactivity magnitude shows a posterior maximum in TDC that is attenuated in FXS, particularly in the occipital region. Peak reactivity frequency varies by region but does not differ significantly between groups. *p < 0.05.

Analysis of the absolute power-derived frequency of maximal changed revealed a marginal main effect of group (*F*(1, 42) = 3.57, *p* =0 .07) and a significant main effect of ROI (*F*(3, 126) = 3.46, *p* = 0.02) (Figure 3), indicating that the absolute peak frequency varied across scalp regions and showed a modest tendency to differ by diagnostic group (Figure 3a). In contrast, sex did not contribute meaningfully to the model (*F(*1, 42) = 0.01, *p* = 0.91). None of the interaction terms reached significance (all *p* > 0.12) (Figure 3b).

#### Absolute Peak Reactivity.

Looking at the magnitude of change at an individual’s most reactive frequency, there were no main effects of group (*F*(1, 42) = 2.09 *p* = 0.16) or sex (*F*(1, 42) = 0.48, *p* = 0.49)(Figure 3c). However, like overall absolute alpha reactivity, a robust main effect of ROI emerged (*F*(3, 126) = 15.99, *p* < 0.001) indicating substantial regional variability in absolute peak reactivity (Figure 3)**.** There was also a significant interaction between group and ROI (*F*(3, 126) = 7.21, *p* < 0.001), indicating that the regional pattern differed between FXS and TDC. No additional interaction terms reached significance (all *p* ≥ 0.98).

Similar to our results for absolute power-derived alpha reactivity, post hoc testing within groups revealed no pairwise differences among ROIs (all *p* ≥ 0.19) in FXS, indicating a largely undifferentiated topography. In contrast, the TDC group showed a pronounced posterior emphasis: occipital reactivity magnitude was significantly greater than frontal (*t* = −6.18, *p* < 0.0001), central (*t* = −6.81, *p* < 0.001), and temporal regions (*t* = −6.96, *p* < 0.001), while no differences were observed among the anterior and temporal sites (all *p* ≥ 0.87).

Between-group comparisons at each ROI further indicated that FXS and TDC differed only at the occipital ROI, where FXS exhibited significantly lower reactivity magnitude than TDC (*t* = −3.97, *p* < 0.001) all other *p* > 0.58).

### Relative Power Measurements

#### Relative Alpha Reactivity.

**Figure 4.**
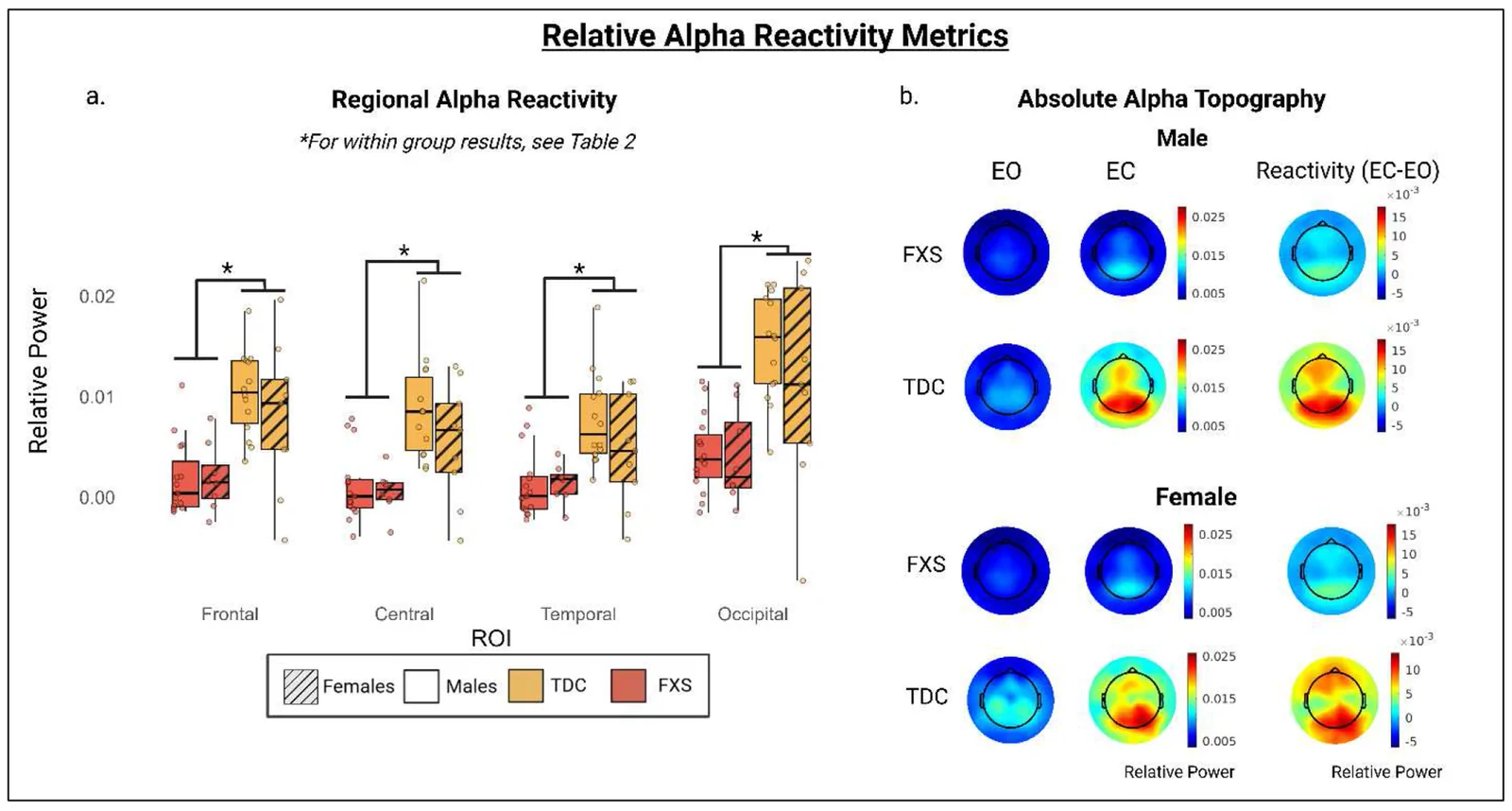
Relative alpha reactivity (8–13 Hz; EC–EO) displayed as (a) boxplots display the median, interquartile range (IQR), with whiskers extending to 1.5× IQR for group and region; individual data points are overlaid, and (b) topographical plots showing the distribution of relative alpha reactivity across the scalp. Both groups demonstrated posterior dominance, but FXS exhibited globally reduced alpha reactivity relative to TDC across all regions, with the largest group differences observed over occipital sites, producing a flatter topographical organization. Detailed within-group post hoc comparisons are summarized in Table 2. *p < 0.05.

Significant main effects of group (*F(*1, 42) = 24.32, *p* < 0.001) and ROI (*F(*3, 126) = 45.95, p < 0.001) indicated that relative power-derived alpha reactivity differed both between diagnostic groups and across brain regions. Importantly, the significant Group × ROI interaction (*F(*3, 126) = 6.78, *p* < 0.001) confirmed that the topographical organization of relative alpha reactivity was not uniform across groups (Figure 4).

Post hoc within-group comparisons showed that both exhibited the expected posterior dominance; in FXS, occipital alpha was significantly higher than frontal (*t* = −3.39, *p* < 0.01), central (*t* = −4.95, *p* < 0.001), and temporal regions (*t* = −4.18, *p* < 0.001) with minimal differentiation among anterior regions (all *p* > 0.40) (Figure 4a, Table 2). In contrast, TDC exhibited a more pronounced regional topography (Figure 4b). Specifically, frontal relative alpha reactivity exceeded both central (*t* = 2.96, *p* = 0.02) and temporal (*t* = 4.37, *p* < 0.001) regions, while the occipital region remained significantly higher than all anterior regions (all *p* < 0.001) (Figure 4b, Table 2).

Between-group contrasts showed that relative alpha reactivity was significantly lower in FXS than in TDC across all regions (frontal, *t* = −4.79, *p* < 0.001; central, *t* = −4.20, *p* < 0.001; temporal, *t* = −3.23, *p* = .002; occipital, *t* = −5.99, *p* < 0.001) (Figure 4a). The largest group difference occurred in the occipital region, followed by frontal and central sites. These results indicate a nonspecific, widespread reduction in relative alpha reactivity in FXS.

#### Relative Peak Frequency.

**Figure 5.**
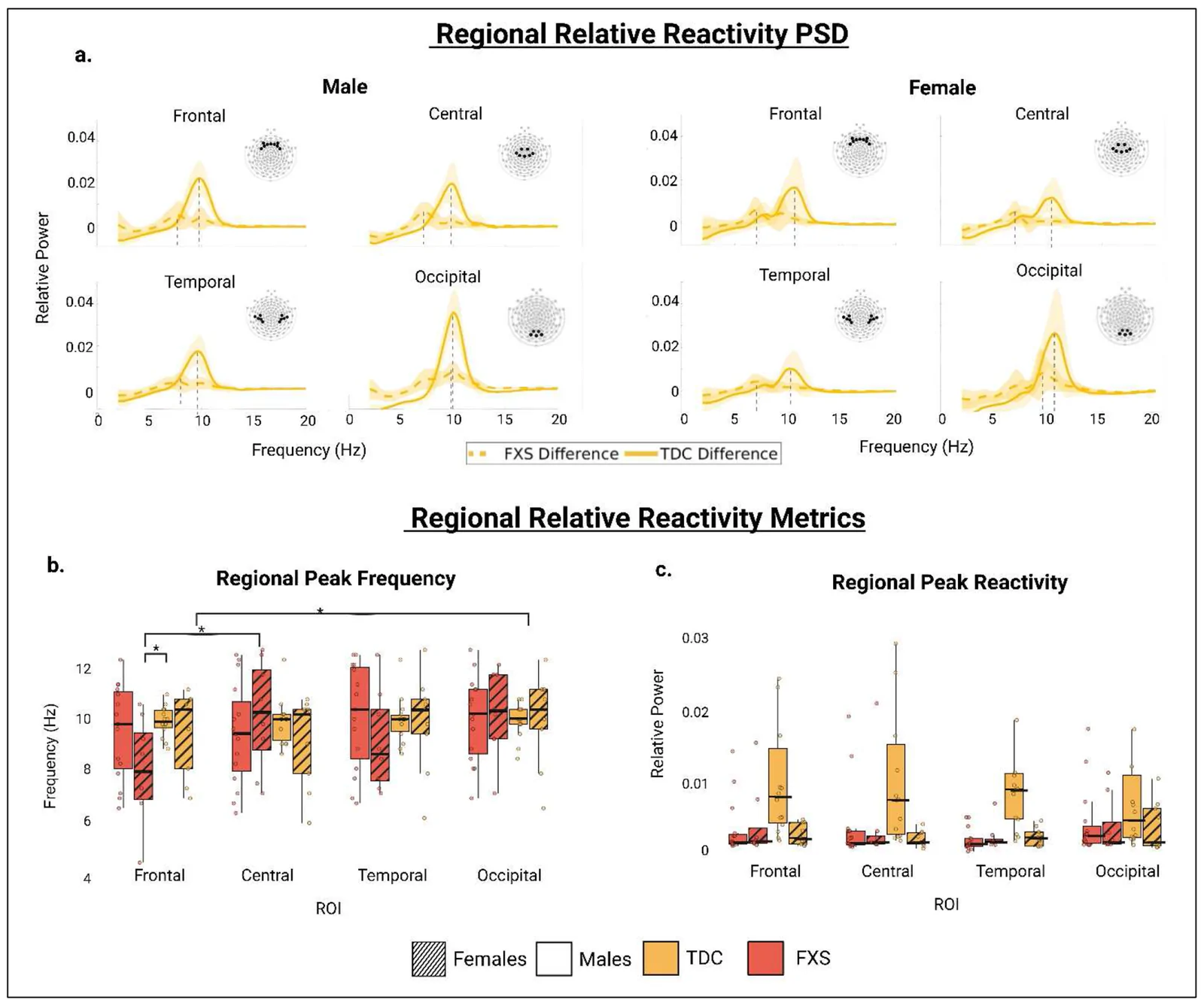
Relative reactivity metrics. Representative PSD plots from cleaned data (a). Relative peak reactivity frequency (b) and peak reactivity magnitude across regions (c), stratified by group and sex. Boxplots show the median, IQR, and 1.5× IQR whiskers with individual data points overlaid. Peak frequency (4–13 Hz range) shows region- and sex-specific variation, with females with FXS exhibiting reduced frontal values relative to central and occipital regions. No significant group differences were observed within regions. Peak reactivity magnitude did not differ significantly by group or region. *p < 0.05.

Looking at the relative power-derived frequency of maximal change (Figure 5a, 5b), there were no main effects of group (*F*(1, 42) = 0.26, *p* = 0.61) or sex (*F*(1, 42) = 0.72, *p* = 0.40), but there was a significant main effect of ROI (*F*(3, 126) = 3.92, *p* = 0.01), indicating regional variation in this measure. In addition, a group × ROI interaction emerged (*F*(3, 126) = 2.72, *p* = 0.05), along with a significant group × ROI × sex interaction (*F*(3, 126) = 3.18, *p* = 0.03), suggesting that group differences in regional patterns further depended on sex.

Post hoc comparisons further clarified the nature of these interactions. Across groups, there were no statistically significant differences between FXS and TDC within any ROI for either females (all *p* ≥ 0.06) or males (all *p* ≥ 0.42) (Figure 5b); however, a trend-level effect was observed in females in the frontal region (*t* = −1.56, *p* = 0.06), suggesting lower relative peak frequency in FXS compared to TDC that did not reach significance. Within-group analyses revealed region-specific patterns that differed by sex, consistent with the three-way interaction. In females with FXS, significant regional differences emerged, with lower relative peak frequency in the frontal region compared to both central (*t* = −3.94, *p* < 0.01) and occipital (*t* = −4.02, *p* < 0.01) regions, while there was not a significant difference in the temporal region (*t* = −2.05, *p* = 0.17).

In contrast, males with FXS, TDC males, and TDC females had no significant differences in relative peak frequency between ROIs (all *p* ≥ 0.31)(Figure 5b). Together, these findings suggest that the observed group × ROI × sex interaction is primarily driven by region-specific alterations in females with FXS, particularly involving reduced values in the frontal region relative to other areas, alongside a trend toward lower frontal values compared to TDC females.

#### Relative Peak Reactivity.

Analysis revealed no main effects of group (*F*(1, 42) = 2.55, *p* = 0.12) or ROI (*F*(3, 126) = 0.73, *p* = 0.54)(Figure 5c). There was a trend level main effect of sex (*F*(1, 42) = 3.14, *p* = 0.08) and a trend level Group × Sex interaction (*F*(1, 42) = 3.45, *p* = 0.07). No other interactions reached significance (all *p* ≥ 0.59).

### Molecular and Clinical Correlations

#### FMRP:

##### Males vs Females.

For both sexes with FXS (n=19, 8 female), no correlations reached statistical significance across alpha reactivity, peak frequency, or reactivity magnitude measures (all *p* > 0.05). However, females with FXS showed a consistent trend of moderate-to-large correlations emerged in the occipital region that may reflect an underlying association with FMRP.

#### FMRP:

##### All Individuals with Measurable FMRP.

In individuals with detectable FMRP expression (n=15, 8 female), a significant negative correlation was observed for relative peak frequency in the frontal region (ρ = −0.55, *p* = .03), such that individuals with more FMRP had a lower relative peak frequency. No other associations between EEG variables and FMRP reached statistical significance across alpha reactivity, peak frequency, or reactivity magnitude measures (all remaining *p* > 0.05)

#### Deviation IQ.

**Figure 6.**
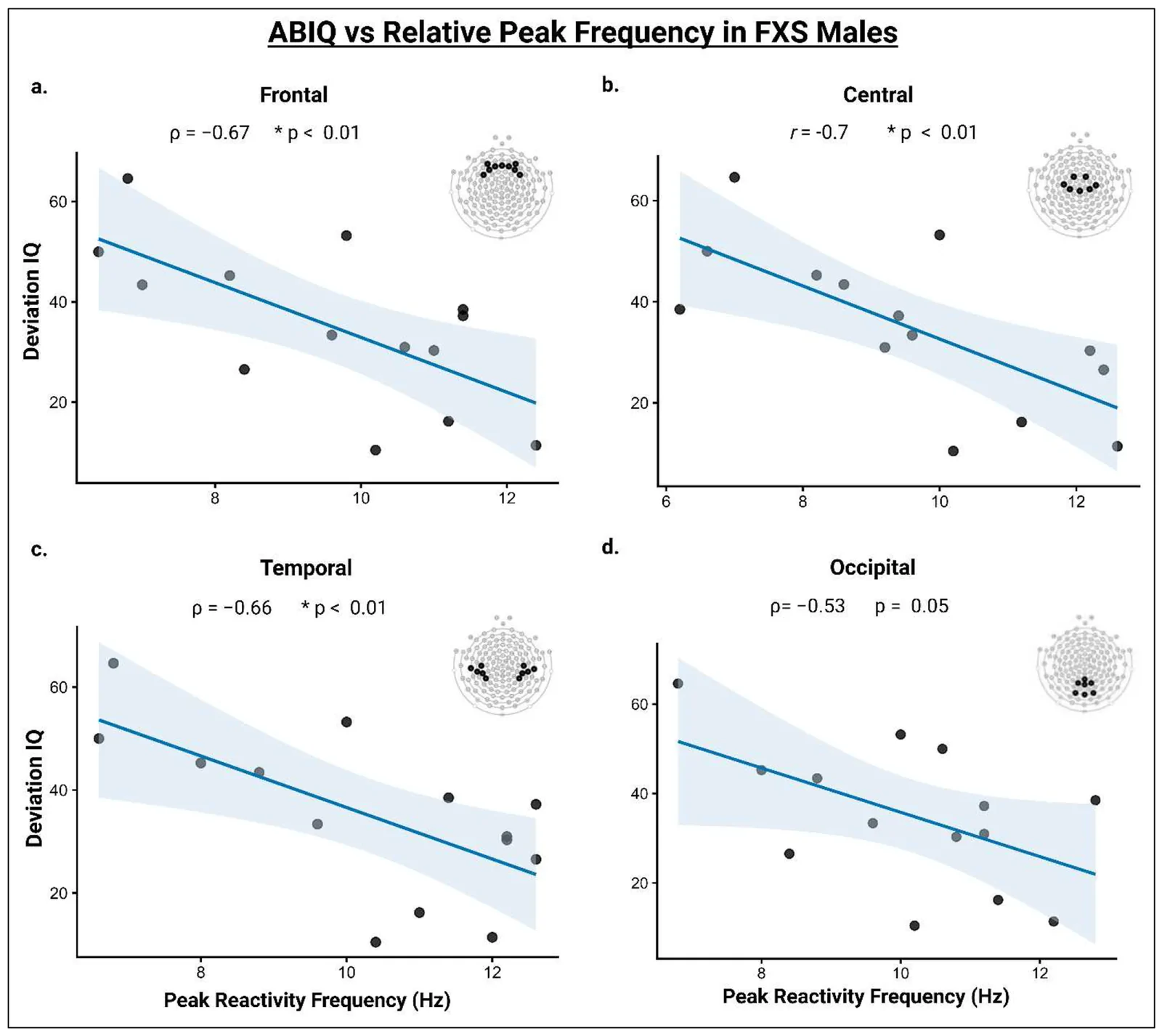
Relationship between relative peak frequency and IQ in males with FXS. Scatterplots illustrating correlations between relative peak reactivity frequency (4–13 Hz) and deviation IQ in males with FXS across cortical regions. Significant negative associations are observed in frontal (a), central (b), and temporal (c) regions, with a trend-level association in the occipital region (d.). Solid lines represent linear fits with shaded regions indicating 95% confidence intervals. *p < 0.05.

EEG measures and IQ were examined separately for males and females. In males, several significant relationships were observed. Absolute alpha reactivity significantly correlated with IQ at central (*r* = 0.60, *p* = 0.02) and temporal sites (*r* = 0.61, *p* = 0.02), suggesting that greater alpha modulation is associated with higher cognitive ability. Relative peak frequency showed robust negative associations with IQ across multiple regions, including central (r = −0.70, *p* < 0.01), frontal (*r* = −0.67, *p* < 0.01), and temporal sites (*r* = −0.66, *p* = .01), with a trend in the occipital region (*r* = −0.53, *p* = 0.05) (Figure 6). This pattern indicates that higher IQ is linked to a lower relative peak frequency, which may reflect a shift toward slower oscillatory dynamics but potentially more efficient or developmentally mature cognitive processing within this population. Reactivity magnitude was also positively associated with IQ in males. Significant effects were observed for absolute magnitude at central (ρ = 0.72, *p* < 0.01, frontal (ρ = 0.76, *p* < 0.01), and temporal regions (ρ = 0.67, *p* = 0.01), as well as for relative magnitude at central (ρ = 0.75, *p* < 0.01), frontal (ρ = 0.61, *p* = .02), and temporal sites (ρ = 0.56, p = .04), indicating that individuals with higher IQ exhibit stronger neural responsiveness on eye closure.

In females, all EEG–IQ associations were nonsignificant (all *p* > .whatever). In the combined sample, EEG–IQ associations were largely nonsignificant, with the exception of a negative correlation between IQ and relative peak frequency in the frontal region (*r* = −0.59, *p* < 0.01), consistent in direction with the male-specific findings.

## Discussion

The present study examined alpha dynamics in FXS by using alpha reactivity as a central measure of interest. Alpha reactivity captures the magnitude of state-dependent EO-to-EC modulation and represents one of the most robust features of alpha physiology. Our analytic framework also identified the theta–alpha frequency showing the largest modulation in response to eye closure, allowing us to test for potential shifts in oscillatory dynamics. By jointly assessing modulation magnitude and peak reactivity frequency across regions, we evaluated whether alpha abnormalities in FXS reflect weakened inhibitory state-dependent modulation, altered oscillator timing, or regionally specific disruptions across distinct alpha systems.

Across both absolute and relative power domains, alpha reactivity was reduced in FXS, though the spatial extent of this reduction differed by measurement approach. Absolute power analyses revealed a localized posterior deficit, with group differences emerging specifically in the occipital region. TDC participants showed robust occipital absolute alpha reactivity consistent with intact sensory inhibitory modulation, whereas individuals with FXS exhibited a flattened alpha topography with minimal posterior specialization. In contrast, relative power analyses demonstrated a widespread attenuation of alpha reactivity across frontal, central, temporal, and occipital regions, with the largest reductions still observed posteriorly. Given prior evidence that elevated theta and aperiodic gamma power index cortical hyperexcitability in FXS[7,11,13], reduced relative alpha reactivity may reflect both diminished inhibitory engagement and a reduced contribution of alpha to overall spectral power. Together, these findings suggest that a focal posterior inhibitory deficit may represent a primary locus of dysfunction that contributes to broader network-level alterations in alpha dynamics.

A key objective of our design was to determine whether altered alpha dynamics in FXS reflect disruption of a single, unitary oscillator or differential impairment across multiple alpha generators and functions. Examination of absolute and relative peak reactivity metrics provided preliminary insight into this question. In the occipital cortex, where alpha power is strongest, peak frequency remained within the canonical alpha range and did not differ significantly between groups despite marked reductions in reactivity magnitude. This pattern was most clear in absolute power–derived measures, which showed a localized posterior deficit in reactivity magnitude with no significant differences in frequency. Together, this pattern is consistent with preserved posterior alpha timing alongside diminished modulatory capacity, suggesting intact intrinsic oscillator dynamics but impaired state-dependent engagement. Given that posterior alpha is predominantly cortico-cortically generated and supports sensory inhibition[15,17,19], these findings point toward a selective weakening of inhibitory modulation within visual networks rather than a fundamental shift in oscillatory frequency. Importantly, this pattern aligns with a broader body of evidence in FXS implicating disrupted inhibitory function, particularly reduced GABAergic regulation and cortical hyperexcitability, suggesting that diminished alpha reactivity may represent another manifestation of impaired inhibitory control rather than a primary deficit in oscillatory generation.

In contrast, alterations observed outside occipital regions were more apparent in relative power–derived measures and are less consistent with a primary disruption of thalamocortical pace making. Instead, they appear to reflect dysfunction within frontal systems supporting executive function and top-down control, particularly in females with FXS. In the present study, females with FXS exhibited reduced frontal peak reactivity frequency relative to central and occipital regions, alongside a trend toward lower frontal values compared to TDC females, whereas no comparable regional effects were observed in males. Notably, these effects emerged in the relative domain, which captures the balance of oscillatory activity within the broader spectral context and may be more sensitive to network-level dysregulation. This female-specific frontal pattern aligns with prior resting-state EEG work in FXS demonstrating reduced frontal alpha connectivity alongside increased gamma connectivity, with both patterns predicting poorer performance on executive function tasks, including increased error rates even after controlling for IQ[46]. Although these findings are derived from resting data rather than task-based paradigms, they suggest that intrinsic alterations in frontal network organizations are behaviorally meaningful and likely reflect compromised executive control systems. Additional studies have also shown disrupted temporal dynamics of frontal alpha activity (e.g., altered burst characteristics), further supporting impaired regulatory coordination within these networks. Within this framework, reduced frontal relative peak frequency may reflect diminished engagement or coordination of executive control networks rather than a global slowing of alpha rhythms[47]. Together, these findings suggest a dissociation in which absolute measures primarily index posterior, sensory-inhibitory dysfunction, whereas relative measures reveal more distributed, network-level alterations involving frontal executive systems, with the latter showing greater sensitivity to sex-specific effects.

To further evaluate the clinical and molecular relevance of these findings, we examined associations between alpha reactivity dynamics and both cognitive functioning and FMRP expression. These analyses revealed a dissociable, sex-dependent pattern, though results should be interpreted cautiously given the exploratory nature of these correlations. In males with FXS, lower relative peak frequency in non-occipital regions was significantly associated with higher IQ, suggesting that variation in relative, network-level alpha reactivity dynamics within frontal and central regions may relate to individual differences in cognitive functioning. Notably, reductions in relative peak frequency imply a shift of the dominant reactive rhythm toward the theta range, which has been previously linked to improved recruitment of distributed networks supporting cognitive control and integration[48–50]. Within this framework, greater theta engagement may reflect adaptive or compensatory mechanisms that support cognition in the context of disrupted alpha-mediated inhibition. In contrast, females did not show significant relationships between relative peak frequency and IQ.

Taken together, these findings point to distinct clinical and molecular correlates of alpha reactivity dynamics that differ by sex and measurement domain. In males, variation in relative, non-posterior measures appears more closely tied to cognitive functioning, potentially reflecting compensatory engagement of slower, theta-linked dynamics within distributed networks. In contrast, in females, absolute posterior measures may remain more sensitive to underlying FMRP expression, suggesting tighter coupling between molecular factors and sensory-inhibitory processes. One possible interpretation is that relatively preserved FMRP levels in females support continued reliance on posterior inhibitory mechanisms, while in males, greater FMRP reduction may necessitate compensatory shifts toward alternative network dynamics. However, given the modest sample size, these interpretations remain tentative. Overall, these results reinforce a non-unitary account of alpha dysfunction in FXS, demonstrating that its clinical and biological correlates diverge across both region and sex, as well as across absolute and relative domains, rather than reflecting a single, global alteration in oscillatory function.

Several limitations should be considered when interpreting these findings. First, the modest sample size, particularly when stratifying by sex, may have reduced statistical power to detect more subtle effects and likely contributed to the presence of trend-level and nonsignificant findings, especially in the molecular and behavioral correlation analyses. Second, relative power measures were calculated separately within each condition (eyes open and eyes closed), which may limit direct comparability of reactivity-related changes across conditions and influence how relative alpha reactivity dynamics are interpreted in relation to underlying spectral composition. Third, the FXS population is characterized by substantial phenotypic, genetic, and behavioral heterogeneity, including variability in FMRP expression, mosaicism status, and cognitive functioning, which may introduce additional variance and obscure more precise brain–behavior relationships. Finally, although all EEG data were acquired in a nominally resting-state paradigm, the primary measure of interest, alpha reactivity, reflects an induced, state-dependent functional change rather than purely intrinsic resting activity. As such, these findings may not be directly comparable to prior studies examining static resting-state power or connectivity and instead reflect the brain’s dynamic capacity for state-dependent modulation. Future studies incorporating larger samples, more homogeneous subgroups, and complementary task-based or naturalistic paradigms will be important for further clarifying the functional significance of these effects.

Despite limitations related to sample size and heterogeneity, the present findings clarify key features of alpha reactivity dynamics in FXS. Alpha reactivity was reduced, but not globally, instead reflecting a combination of localized posterior deficits and broader network-level alterations. These results support the presence of multiple, functionally distinct alpha systems rather than a single, unitary disturbance, and provide no evidence for uniform slowing of alpha dynamics. Importantly, effects varied by sex, indicating that the relationship between neural dynamics, cognition, and molecular factors differs across individuals with FXS. Overall, these findings support a multi-system model of alpha dysfunction in FXS and highlight alpha reactivity dynamics as a promising translational marker of both inhibitory and network-level abnormalities.

## Figures and Tables

**Table 1.** Participant Demographic Information

*Measure*	FXS (n = 23; 8 female)			TDC (n = 23; 9 female)		
	*n*	Mean (SD)	Range	*n*	Mean (SD)	Range
		
Age (years)	23	30.18 (7.99)	17-44	23	27.00 (7.43)	15-43
		
SB5 Deviation IQ (Abbreviated)						
Overall	22	49 (28)	10-105	23	108 (10)	91-130
Male	14	35 (15)	10-64	14	109 (10)	97-130
Female	8	74 (28)	22-105	9	105 (12)	91-121
		
FMRP						
Overall	19	11.24 (14.12)	0.07-39.85	-	-	-
Male	11	0.84 (1.11)	0.07-3.48	-	-	-
Female	8	25.54 (10.36)	5.83-39.85	-	-	-
		
Race						
White	20	-	-	21	-	-
Asian	0	-	-	1	-	-
Multiracial	1	-	-	1	-	-
Other	1	-	-	0	-	-
Prefer not to say	1	-	-	0	-	-
		
Ethnicity						
Hispanic or Latino	1	-	-	0	-	-
Not Hispanic or Latino	22	-	-	23	-	-

SD, standard deviation; n, sample size; FMRP, Fragile X messenger ribonucleoprotein;

**Table 2.** Relative alpha reactivity: Post-hoc within-group results from Figure 4a.

*Contrast*	FXS (n = 23; 8 female)			
	Male		Female	
		
	t(126)	*p*	t(126)	*p*
Frontal–Central	0.92	0.794	1.26	0.591
Frontal–Temporal	0.71	0.894	0.46	0.967
Frontal–Occipital	**−3.1**	**0.012**	−1.92	0.223
Central–Temporal	−0.21	0.997	−0.8	0.856
Central–Occipital	**−4.03**	**<0.001**	**−3.18**	**0.01**
Temporal–Occipital	**−3.81**	**0.001**	−2.39	0.085
	
*Contrast*	THC (n = 23; 9 female)			
	Male		Female	
		
	t(126)	*p*	t(126)	*p*
Frontal–Central	1.86	0.25	2.31	0.102
Frontal–Temporal	**3.2**	**0.009**	**3.04**	**0.015**
Frontal–Occipital	**−5.63**	**<0.001**	**−3.44**	**0.004**
Central–Temporal	1.33	0.543	0.74	0.883
Central–Occipital	**−7.49**	**<0.001**	**−5.75**	**<0.001**
Temporal–Occipital	**−8.83**	**<0.001**	**−6.48**	**<0.001**

n, sample size; t, t-value; *p*, p-value. Bolded text indicates a statically significant result (*p*<0.05).

## Data Availability

The data that support the findings of this study are available from the corresponding author (S.D.) upon reasonable request.

## References

[1] Yu S. Fragile X Genotype Characterized by an Unstable Region of DNA. Science 252, 1179–1181 (1991). 2031189 10.1126/science.252.5009.1179

[2] Darnell J. C. FMRP Stalls Ribosomal Translocation on mRNAs Linked to Synaptic Function and Autism. Cell 146, 247–261 (2011). 21784246 10.1016/j.cell.2011.06.013 PMC3232425

[3] Richter J. D. & Zhao X. The molecular biology of FMRP: new insights into fragile X syndrome. Nat. Rev. Neurosci. 22, 209–222 (2021). 33608673 10.1038/s41583-021-00432-0 PMC8094212

[4] Genovese A. C. & Butler M. G. Systematic Review: Fragile X Syndrome Across the Lifespan with a Focus on Genetics, Neurodevelopmental, Behavioral and Psychiatric Associations. Genes 16, 149 (2025). 40004478 10.3390/genes16020149 PMC11855108

[5] Lachiewicz A. M. Sensory Symptoms and Signs of Hyperarousal in Individuals with Fragile X Syndrome: Findings from the FORWARD Registry and Database Multisite Study. J. Autism Dev. Disord. 54, 4259–4277 (2024). 37840096 10.1007/s10803-023-06135-y PMC11461590

[6] Tassone F. FMR1 CGG allele size and prevalence ascertained through newborn screening in the United States. Genome Med. 4, 100 (2012). 23259642 10.1186/gm401 PMC4064316

[7] Pedapati E. V. Neocortical localization and thalamocortical modulation of neuronal hyperexcitability contribute to Fragile X Syndrome. Commun. Biol. 5, 442 (2022). 35546357 10.1038/s42003-022-03395-9 PMC9095835

[8] Pedapati E. THALAMOCORTICAL DYSRHYTHMIA AS A UNIFYING MODEL OF NEUROPSYCHIATRIC AND NEUROSENSORY DYSFUNCTION IN FRAGILE X SYNDROME. J. Am. Acad. Child Adolesc. Psychiatry 61, S275–S276 (2022).

[9] Ethridge L. E. Auditory EEG Biomarkers in Fragile X Syndrome: Clinical Relevance. Front. Integr. Neurosci. 13, 60 (2019). 31649514 10.3389/fnint.2019.00060 PMC6794497

[10] Miyakoshi M. Hyper-extralemniscal model of Fragile X syndrome. Cereb. Cortex 35, bhaf141 (2025). 40518965 10.1093/cercor/bhaf141 PMC12167856

[11] Wang J. A resting EEG study of neocortical hyperexcitability and altered functional connectivity in fragile X syndrome. J. Neurodev. Disord. 9, 11 (2017). 28316753 10.1186/s11689-017-9191-z PMC5351111

[12] Takarae Y., Zanesco A., Erickson C. A. & Pedapati E. V. EEG Microstates as Markers for Cognitive Impairments in Fragile X Syndrome. Brain Topogr. 37, 432–446 (2024). 37751055 10.1007/s10548-023-01009-z

[13] Smith E. G. Sex differences in resting EEG power in Fragile X Syndrome. J. Psychiatr. Res. 138, 89–95 (2021). 33836434 10.1016/j.jpsychires.2021.03.057 PMC8192450

[14] Baek K. EEG alpha reactivity in cognitive aging and dementia: clinical implications and cholinergic mechanisms. Front. Hum. Neurosci. 20, 1665301 (2026). 41834908 10.3389/fnhum.2026.1665301 PMC12982453

[15] Pascucci D. EEG brain waves and alpha rhythms: Past, current and future direction. Neurosci. Biobehav. Rev. 176, 106288 (2025). 40669525 10.1016/j.neubiorev.2025.106288

[16] Klimesch W. Alpha-band oscillations, attention, and controlled access to stored information. Trends Cogn. Sci. 16, 606–617 (2012). 23141428 10.1016/j.tics.2012.10.007 PMC3507158

[17] Klimesch W., Sauseng P. & Hanslmayr S. EEG alpha oscillations: The inhibition–timing hypothesis. Brain Res. Rev. 53, 63–88 (2007). 16887192 10.1016/j.brainresrev.2006.06.003

[18] Llins R. R., Ribary U., Jeanmonod D., Kronberg E. & Mitra P. P. Thalamocortical dysrhythmia: A neurological and neuropsychiatric syndrome characterized by magnetoencephalography. Proc. Natl. Acad. Sci. 96, 15222–15227 (1999). 10611366 10.1073/pnas.96.26.15222 PMC24801

[19] Halgren M. The generation and propagation of the human alpha rhythm. Proc. Natl. Acad. Sci. 116, 23772–23782 (2019). 31685634 10.1073/pnas.1913092116 PMC6876194

[20] Hindriks R. & Van Putten M. J. A. M. Thalamo-cortical mechanisms underlying changes in amplitude and frequency of human alpha oscillations. NeuroImage 70, 150–163 (2013). 23266701 10.1016/j.neuroimage.2012.12.018

[21] Vijayan S. & Kopell N. J. Thalamic model of awake alpha oscillations and implications for stimulus processing. Proc. Natl. Acad. Sci. 109, 18553–18558 (2012). 23054840 10.1073/pnas.1215385109 PMC3494906

[22] Pfeiffer B. E. & Huber K. M. Fragile X Mental Retardation Protein Induces Synapse Loss through Acute Postsynaptic Translational Regulation. J. Neurosci. 27, 3120–3130 (2007). 17376973 10.1523/JNEUROSCI.0054-07.2007 PMC6672463

[23] Patel A. B., Loerwald K. W., Huber K. M. & Gibson J. R. Postsynaptic FMRP Promotes the Pruning of Cell-to-Cell Connections among Pyramidal Neurons in the L5A Neocortical Network. J. Neurosci. 34, 3413–3418 (2014). 24573297 10.1523/JNEUROSCI.2921-13.2014 PMC3935093

[24] He C. X. & Portera-Cailliau C. The trouble with spines in fragile X syndrome: density, maturity and plasticity. Neuroscience 251, 120–128 (2013). 22522472 10.1016/j.neuroscience.2012.03.049 PMC3422423

[25] Ordemann G. J., Lyuboslavsky P., Kizimenko A. & Brumback A. C. Fmr1 KO causes delayed rebound spike timing in mediodorsal thalamocortical neurons through regulation of HCN channel activity. Preprint at 10.1101/2025.02.02.636122 (2025).

[26] Barry R. J. & De Blasio F. M. EEG differences between eyes-closed and eyes-open resting remain in healthy ageing. Biol. Psychol. 129, 293–304 (2017). 28943465 10.1016/j.biopsycho.2017.09.010

[27] Isler J. R. Longitudinal characterization of EEG power spectra during eyes open and eyes closed conditions in children. Psychophysiology 60, e14158 (2023). 35968705 10.1111/psyp.14158 PMC9729391

[28] Fló E. Predicting attentional focus: Heartbeat-evoked responses and brain dynamics during interoceptive and exteroceptive processing. PNAS Nexus 3, pgae531 (2024). 39677366 10.1093/pnasnexus/pgae531 PMC11645458

[29] Marx E. Eye closure in darkness animates sensory systems. NeuroImage 19, 924–934 (2003). 12880821 10.1016/s1053-8119(03)00150-2

[30] Petro N. M. Eyes-closed versus eyes-open differences in spontaneous neural dynamics during development. NeuroImage 258, 119337 (2022). 35636737 10.1016/j.neuroimage.2022.119337 PMC9385211

[31] Wan L. From eyes-closed to eyes-open: Role of cholinergic projections in EC-to-EO alpha reactivity revealed by combining EEG and MRI. Hum. Brain Mapp. 40, 566–577 (2019). 30251753 10.1002/hbm.24395 PMC6338213

[32] Flanagan D. P. & McDonough E. M. Contemporary Intellectual Assessment: Theories, Tests, and Issues. (The Guilford Press, New York, 2022).

[33] Sansone S. M. Improving IQ measurement in intellectual disabilities using true deviation from population norms. J. Neurodev. Disord. 6, 16 (2014). 26491488 10.1186/1866-1955-6-16 PMC4613563

[34] Boggs A. E. Optimization, validation and initial clinical implications of a Luminex-based immunoassay for the quantification of Fragile X Protein from dried blood spots. Sci. Rep. 12, 5617 (2022). 35379866 10.1038/s41598-022-09633-8 PMC8980090

[35] Ethridge L. E. Validating brain activity measures as reliable indicators of individual diagnostic group and genetically mediated sub-group membership in Fragile X Syndrome. Sci. Rep. 14, 22982 (2024). 39362936 10.1038/s41598-024-72935-6 PMC11450163

[36] Delorme A. & Makeig S. EEGLAB: an open source toolbox for analysis of single-trial EEG dynamics including independent component analysis. J. Neurosci. Methods 134, 9–21 (2004). 15102499 10.1016/j.jneumeth.2003.10.009

[37] Mullen T. R. Real-time neuroimaging and cognitive monitoring using wearable dry EEG. IEEE Trans. Biomed. Eng. 62, 2553–2567 (2015). 26415149 10.1109/TBME.2015.2481482 PMC4710679

[38] Kothe C. A. & Makeig S. BCILAB: a platform for brain–computer interface development. J. Neural Eng. 10, 056014 (2013). 23985960 10.1088/1741-2560/10/5/056014

[39] Miyakoshi M. Artifact subspace reconstruction: a candidate for a dream solution for EEG studies, sleep or awake. SLEEP 46, zsad241 (2023). 37715954 10.1093/sleep/zsad241 PMC10710985

[40] Bellato A., Arora I., Kochhar P., Hollis C. & Groom M. J. Atypical Electrophysiological Indices of Eyes-Open and Eyes-Closed Resting-State in Children and Adolescents with ADHD and Autism. Brain Sci. 10, 272 (2020). 32370023 10.3390/brainsci10050272 PMC7288160

[41] Wilkinson C. L. & Nelson C. A. Increased aperiodic gamma power in young boys with Fragile X Syndrome is associated with better language ability. Mol. Autism 12, 17 (2021). 33632320 10.1186/s13229-021-00425-x PMC7908768

[42] Bates D., Maechler M., Bolker B. & Walker S. Fitting Linear Mixed-Effects Models Using {lme4}. 67, 1–48 (2015).

[43] Lenth R. V. & Piaskowski J. emmeans: Estimated Marginal Means, aka Least-Squares Means. https://rvlenth.github.io/emmeans/ (2026).

[44] Wickham H. Ggplot2: Elegant Graphics for Data Analysis. (Springer-Verlag New York, 2016).

[45] Biorender. BioRender Inc.

[46] Schmitt L. M. Altered frontal connectivity as a mechanism for executive function deficits in fragile X syndrome. Mol. Autism 13, 47 (2022). 36494861 10.1186/s13229-022-00527-0 PMC9733336

[47] Norris J. E. Hemispheric Utilization of Alpha Oscillatory Dynamics as a Unique Biomarker of Neural Compensation in Females with Fragile X Syndrome. ACS Chem. Neurosci. 13, 3389–3402 (2022). 36411085 10.1021/acschemneuro.2c00404 PMC10199731

[48] Adamczyk A. K. & Wyczesany M. Theta-band Connectivity within Cognitive Control Brain Networks Suggests Common Neural Mechanisms for Cognitive and Implicit Emotional Control. J. Cogn. Neurosci. 35, 1656–1669 (2023). 37584600 10.1162/jocn_a_02034

[49] Senoussi M. Theta oscillations shift towards optimal frequency for cognitive control. Nat. Hum. Behav. 6, 1000–1013 (2022). 35449299 10.1038/s41562-022-01335-5

[50] Gómez-Lombardi A. The cognitive triad network - oscillation - behaviour links individual differences in EEG theta frequency with task performance and effective connectivity. Sci. Rep. 14, 21482 (2024). 39277643 10.1038/s41598-024-72229-x PMC11401920

